# Chronic Deficiency of Nitric Oxide Affects Hypoxia Inducible Factor-1α (HIF-1α) Stability and Migration in Human Endothelial Cells

**DOI:** 10.1371/journal.pone.0029680

**Published:** 2011-12-27

**Authors:** Maria Grazia Cattaneo, Elisa Cappellini, Roberta Benfante, Maurizio Ragni, Fausta Omodeo-Salè, Enzo Nisoli, Nica Borgese, Lucia M. Vicentini

**Affiliations:** 1 Department of Pharmacology, School of Medicine, Università degli Studi di Milano, Milano, Italy; 2 CNR, Institute of Neuroscience, Milano, Italy; 3 Department of Molecular Sciences Applied to Biosystems, Faculty of Pharmacy, Università degli Studi di Milano, Milano, Italy; 4 Department of Pharmacobiological Science, University of Catanzaro “Magna Graecia”, Roccelletta di Borgia (Cz), Italy; University of Colorado Denver, United States of America

## Abstract

**Background:**

Endothelial dysfunction in widely diffuse disorders, such as atherosclerosis, hypertension, diabetes and senescence, is associated with nitric oxide (NO) deficiency. Here, the behavioural and molecular consequences deriving from NO deficiency in human umbilical vein endothelial cells (HUVECs) were investigated.

**Results:**

Endothelial nitric oxide synthase (eNOS) was chronically inhibited either by *N* ^G^-Nitro-l-arginine methyl ester (l-NAME) treatment or its expression was down-regulated by RNA interference. After long-term l-NAME treatment, HUVECs displayed a higher migratory capability accompanied by an increased Vascular Endothelial Growth Factor (VEGF) and VEGF receptor-2 (kinase insert domain receptor, KDR) expression. Moreover, both pharmacological and genetic inhibition of eNOS induced a state of pseudohypoxia, revealed by the stabilization of hypoxia-inducible factor-1α (HIF-1α). Furthermore, NO loss induced a significant decrease in mitochondrial mass and energy production accompanied by a lower O_2_ consumption. Notably, very low doses of chronically administered DETA/NO reverted the HIF-1α accumulation, the increased VEGF expression and the stimulated migratory behaviour detected in NO deficient cells.

**Conclusion:**

Based on our results, we propose that basal release of NO may act as a negative controller of HIF-1α levels with important consequences for endothelial cell physiology. Moreover, we suggest that our experimental model where eNOS activity was impaired by pharmacological and genetic inhibition may represent a good *in vitro* system to study endothelial dysfunction.

## Introduction

Integrity of endothelial cells is crucial for the maintenance of vascular homeostasis. The endothelium explicates its physiological functions by producing active molecules, among which nitric oxide (NO) is particularly important. By diffusing into neighboring smooth muscle cells, endothelial-produced NO induces vasorelaxation, thereby controlling blood pressure levels [1], [2]. NO generated in the endothelium also has antiaggregant activity that protects the cardiovascular system from thrombosis and acute events [2].

Consistent with the key role of this gaseous messenger in cardiovascular physiology, NO loss is a dangerous event that is associated with endothelial dysfunction typical of diffuse pathological conditions like atherosclerosis and senescence [3]–[5]. Moreover, the deficiency of NO and endothelial nitric oxide synthase (eNOS) activity is thought to be crucial for the development and/or acceleration of the important vascular complications associated with diabetes [6].

In addition to its effect on smooth muscle cells and platelets, NO generated by the endothelium has important functions in the endothelial cells (ECs) themselves. Indeed, the gaseous messenger plays a key role in the process of angiogenesis, stimulating proliferation, migration and differentiation of ECs to form new blood vessels [7]. In particular, NO acutely produced by angiogenic factors, such as Vascular Endothelial Growth Factor (VEGF) [8]–[10], endothelin [11], substance P [12] and oxytocin [13] is crucial for stimulation of EC migration.

Together with the stimulatory effect of acute NO on EC chemotaxis, also the concentration and timing of NO release appear to be of crucial importance in determining the final outcome on EC physiology. In particular, recent work from our laboratory has demonstrated that long term inhibition of eNOS in Human Umbilical Vein ECs (HUVECs) by exposure to the NOS inhibitor N^G^-Nitro-L-arginine methyl ester (L-NAME), increases the migratory behaviour of these cells in Boyden chambers assays carried out immediately after removal of the drug [14]. These results suggest that basal NO, at variance with the gas released acutely in response to motogenic factors, diminishes the migratory ability of ECs. The tonic inhibitory effect of basal NO on migration, by acting as a brake on inappropriate migration, could prevent exaggerated angiogenic responses and thus be an important homeostatic factor in EC physiology.

In the present study, we have further investigated the effects of chronic NO deprivation on EC physiology, and attempted to unravel the pathway linking basal NO to migratory ability. Results obtained both by long term pharmacological inhibition and by genetic silencing of eNOS indicate that NO loss induces profound modifications in EC physiology, leading to a general decrease of mitochondrial mass and metabolic activity, to an accumulation of Hypoxia Inducible Factor-1α (HIF-1α) in normoxia and to enhanced chemotactic migration as a consequence of the increased HIF-1α levels. These results have important implication for our understanding of the consequences of NO deprivation in cardiovascular pathology.

## Results

### HUVECs chronically treated with L-NAME are not apoptotic, but have decreased mitochondrial mass and function

**Figure 1:**
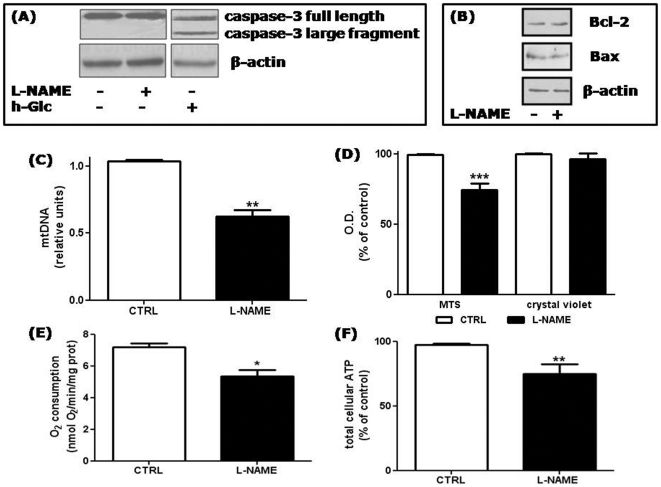
Effect of chronic NO deprivation on HUVEC vitality and mitochondrial mass and function. (A) Lysates of HUVECs treated for 48 h with 5 mM L-NAME or 30 mM glucose (high glucose, h-Glc) were separated by 12% SDS-PAGE and immunoblotted with an anti-caspase 3 antibody which recognized full length caspase-3 (35 kDa) and its large fragment resulting from cleavage (17 kDa). β-actin was used as a loading control. Shown is a representative blot of 2 comparable experiments. (B) Total cell lysates prepared as described in (A) were separated by SDS-PAGE and immunoblotted with anti Bcl–2 or anti Bax antibodies. β-actin was used as a loading control. Shown is a representative blot of 2 comparable experiments. (C) Mitochondrial DNA (mtDNA) was quantified by RT-qPCR from control cells (CTRL) or from cells treated with L-NAME for 48 h, and normalized to the level of the housekeeping gene 18S. **p<0.01; *t* test; n = 3. (D) Mitochondrial activity of control and L-NAME treated cells was evaluated by means of MTS. In parallel samples, the total cell number was measured by crystal violet staining. ***p<0.001 *vs* control cells (CTRL, set at 100%); *t* test; n = 7. (E) After L-NAME treatment, oxygen consumption was reduced by 25±6% in comparison to control cells. The values were normalized to the cell protein content. *p<0.05; *t* test, n = 3. (F) Total cellular ATP levels were reduced by 25±7% after 48 h of 5 =  mM L-NAME treatment. **p<0.01 *vs* control cells (CTRL, set at 100%); *t* test; n = 8.

To characterize the effects of long term NO deprivation on human ECs, we first analyzed possible changes in cell viability. As shown in **Figure 1A**, treatment with L-NAME for 48 h did not induce caspase-3 cleavage, which instead occurred when HUVECs were exposed to high glucose (30 mM for 48 h), a condition known to be apoptotic for these cells [15]. Moreover, quantification of apoptosis/necrosis by annexin V-conjugated FITC and PI staining followed by FACS analysis did not show any difference in the apoptotic index between control and L-NAME treated HUVECs (0.16±0.03 and 0.15±0.05 in control and L-NAME treated cells, respectively). Also the percentage of necrotic cells was unaffected by the treatment, ranging from 8.3±0.26% in control cells to 4.1±0.21% in cells treated with L-NAME. Finally, we checked the levels of Bcl-2 and Bax, well-known proteins involved in the regulation of apoptosis endowed with anti-apoptotic and pro-apoptotic activity respectively, and found that their expression was unchanged by L-NAME treatment (**Figure 1B**).

In HeLa cells and adipocytes, basal NO enhances mitochondrial mass [16], [17], however the effect of the gas on mitochondrial biogenesis in ECs had not been investigated. As shown in **Figure 1C**, HUVECs treated with L-NAME for 48 h showed a decreased amount of mitochondrial DNA (mtDNA) as compared to untreated cells (by 38±0.05%). Moreover, incorporation of the metabolic indicator MTS was lower in L-NAME treated cells (with a mean reduction of 26±5%) (**Figure 1D**) indicating a decreased mitochondrial activity in NO deficient cells as compared to control cells. In agreement, oxygen consumption as well as ATP levels were reduced in the L-NAME treated cells (**Figures 1E and F**)**.**

### The NO donor DETA-NO completely reverts the increased migratory behavior induced in HUVECs by long-term L-NAME treatment

**Figure 2:**
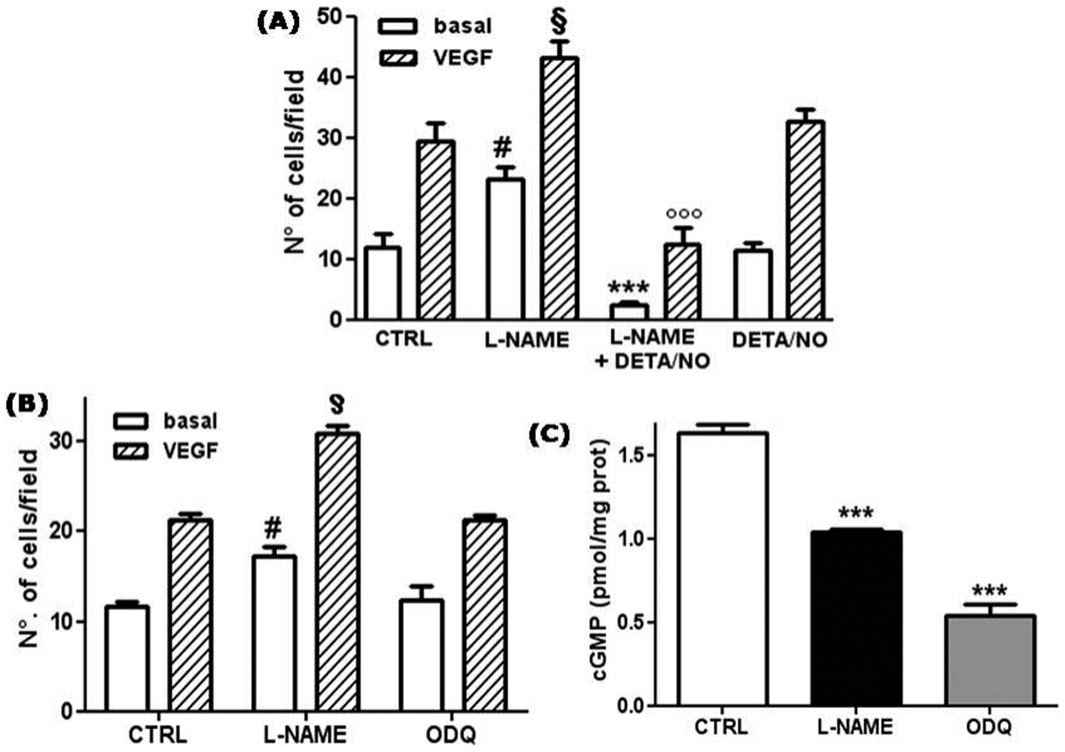
The enhancement in HUVEC migration induced by L-NAME is reverted by the NO donor DETA-NO and is independent of the cGMP pathway. (A) HUVECs were treated for 48 h with 5 mM L-NAME in the absence or in the presence of 500 nM DETA/NO for the last 24 h, as indicated. Chemotaxis experiments were then performed using 25 ng/ml VEGF as attractants. Results are expressed as the number of migrating cells. #p<0.001 *vs* basal migration in control cells (CTRL); §p<0.01 *vs* VEGF-induced migration in control cells; ***p<0.001 *vs* basal migration in L-NAME treated cells; °°°p<0.001 *vs* VEGF-induced migration in L-NAME treated cells; no significant differences between control and DETA/NO treated cells (One-way ANOVA with Bonferroni's test, n = 15). (B) HUVECs were treated for 48 h with 5 mM L-NAME or 1 µM ODQ, and chemotaxis experiments were performed as described in (A). Results are expressed as the number of migrating cells in the different experimental conditions. #p<0.001 *vs* basal migration in control cells (CTRL); §p<0.001 *vs* VEGF-induced migration in control cells; no significant differences between control and ODQ treated cells (One-way ANOVA with Bonferroni's test, n = 3). (C) cGMP accumulation in HUVECs treated for 48 h with L-NAME or ODQ was evaluated by EIA and expressed as pmol of cGMP normalized to the cell protein content (pmol/mg protein). ***p<0.001; One-way ANOVA with Bonferroni's test; n = 3.

Our previous results demonstrated that long-term treatment of HUVECs with L-NAME induced a strong increase of the cell migratory capacity assayed in Boyden chambers [14]. These results suggested that chronic, constitutive NO production exerts a tonic inhibition on HUVEC migratory behavior. To validate this hypothesis, we investigated whether long-term administration of the slow NO donor DETA-NO would reverse the L-NAME-enhanced migration of HUVECs. As shown in **Figure 2A**, chronic treatment of HUVECs with L-NAME for 48 h was confirmed to increase the cell migratory response to VEGF by about 45%. Notably, also basal migration was augmented in a similar manner. No significant increase in cell migratory behavior was observed after 24 h treatment with L-NAME (data not shown). A dose as low as 500 nM of DETA-NO, supplied to the L-NAME treated cells for the last 24 h of the treatment, completely abolished the increased migration observed in both basal and VEGF-stimulated NO deficient HUVECs, thus confirming that enhanced HUVEC migration caused by L-NAME was due to NO deprivation. Notably, cells treated with the combination of L-NAME and DETA-NO showed diminished migration compared to cells treated with the donor alone. This result can probably be explained by a biphasic effect of chronic NO, as demonstrated for HeLa cells in our previous work [14].

To investigate whether the increased migratory behavior caused by chronic NOS inhibition was due to a deficiency in cyclic GMP (cGMP) consequent to NO deprivation, we evaluated the effect of long-term treatment with the guanylate cyclase inhibitor ODQ on HUVEC migration in Boyden chambers. After treatment of the cells for 48 h either with L-NAME or with ODQ, migration assays were performed in the absence of the drugs. As shown in **Figure 2B**, chronic treatment with ODQ did not induce any change either in basal or in VEGF-stimulated HUVEC motility. The measurement of cGMP accumulation in HUVECs chronically treated with L-NAME or ODQ confirmed the ability of both drugs to significantly reduce cGMP levels (**Figure 2C**). These results indicated that blunting of the cGMP signaling pathway is not involved in the stimulatory effect induced by NO depletion.

### Effects of long term L-NAME treatment on eNOS, VEGF, KDR expression and VEGF signaling in HUVECs

**Figure 3:**
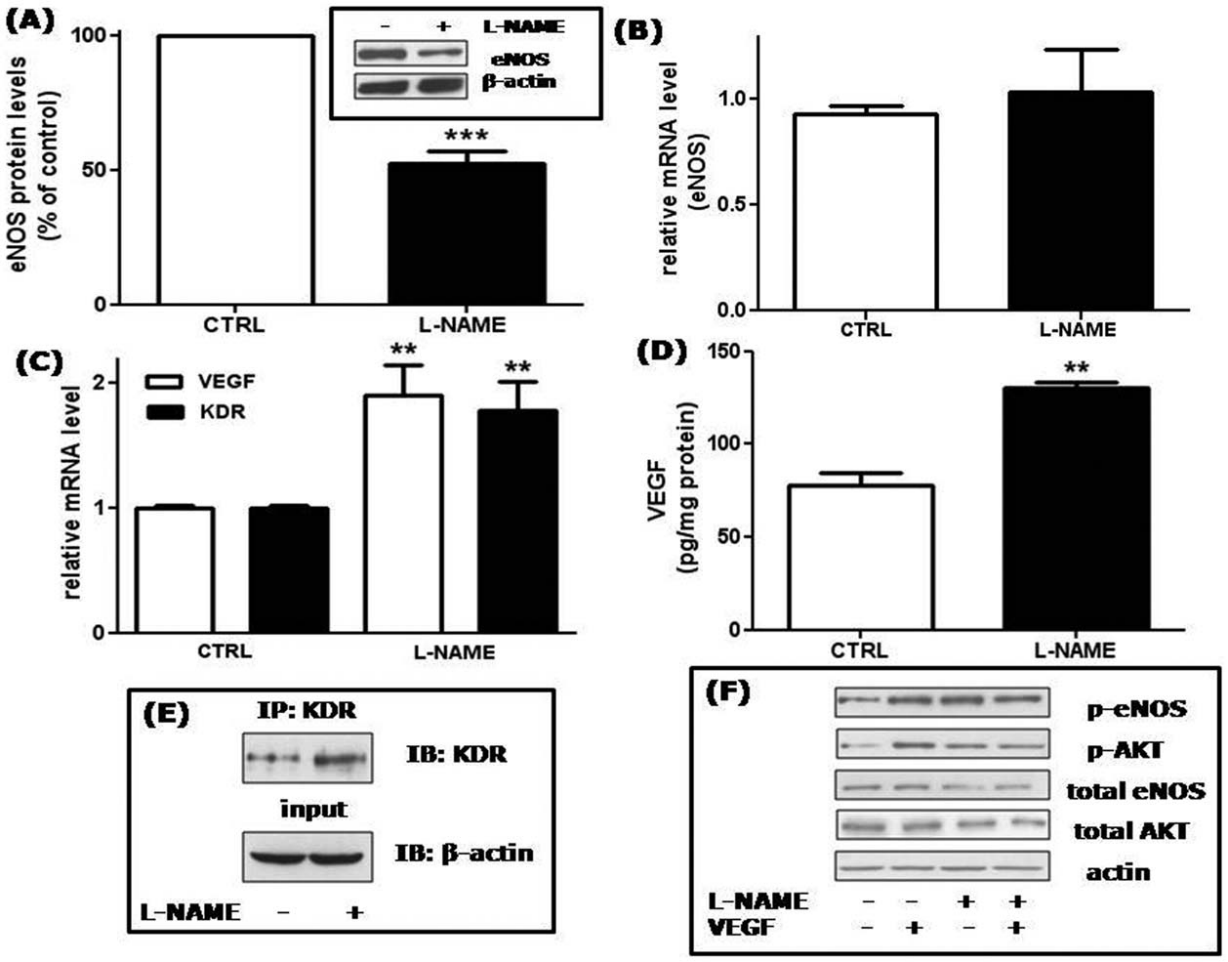
Effects of L-NAME treatment on eNOS, VEGF and KDR expression. (A) Densitometric analysis of eNOS protein expression. ***p<0.001; *t* test; n = 11. Inset: a representative blot out of eleven is shown. Total eNOS protein was evaluated by western blotting on lysates prepared from control cells (lane 1) or from 48 h L-NAME treated cells (lane 2). β-actin was used as a loading control. (B) eNOS RNA levels were measured by RT-qPCR and normalized to the level of the housekeeping gene 18S. No significant differences between control and L-NAME treated cells (*t* test, n = 3). (C) VEGF and KDR RNA levels were measured by RT-qPCR and normalized to the level of the housekeeping gene 18S. **p<0.01 *vs* control cells (CTRL); *t* test; n = 6−4 for VEGF and KDR, respectively. (D) VEGF protein levels were detected by ELISA measurement in conditioned media collected from control or 48 h L-NAME treated cells. Results are expressed as pg of VEGF normalized to the cell protein content (pg/mg protein). **p<0.01; *t* test; n = 3. (E) KDR protein was visualized by western blot after immunoprecipitation with KDR antibodies of HUVEC lysates obtained from control (lane 1) or from 48 h L-NAME treated cells (lane 2). An aliquot of total cell lysates was immunoblotted with β-actin antibodies as a control (input). Shown is a representative blot of 2 comparable experiments. (F) Control cells (lanes 1 and 2) or 48 h L-NAME treated cells (lanes 3 and 4) were stimulated for 5 min with 25 ng/ml VEGF. Aliquots of cell lysates were separated by 10% SDS-PAGE and immunoblotted with the indicated antibodies. Actin was used as a loading control. Shown is a representative blot of 4 comparable experiments.

We previously considered the hypothesis that the improved migratory ability of HUVECs chronically treated with L-NAME could be caused by a rebound effect: expression of eNOS in response to its chronic inhibition could have been increased, thus stimulating migration *via* enhanced NO release upon exposure of the cells to motogenic factors in the absence of the inhibitor. Our analysis, however, suggested that eNOS was not increased by chronic L-NAME treatment [14]. To investigate this phenomenon further, we quantitatively analyzed both eNOS protein and mRNA levels in treated and untreated cells. As shown in **Figure 3A**, a significant decrease in eNOS protein levels (by 48±5%) was detected in treated cells. In contrast, no variation in the eNOS mRNA level, measured by RT-qPCR, was observed (1.04±0.3 fold in comparison to untreated cells) (**Figure 3B**), suggesting that chronic inhibition of eNOS causes an increased degradation of the enzyme and/or impairment of the translation of its mRNA.

In an attempt to explain the mechanism through which NO deprivation enhances migration, we investigated how chronic L-NAME treatment affects the expression levels of the VEGF receptor-2 (kinase insert domain receptor, KDR). We also analyzed VEGF itself, as endogenous production of the growth factor could potentiate migration by an autocrine loop. RT-qPCR analysis demonstrated that both VEGF and KDR mRNA levels increased, 1.91±0.2 and 1.79±0.2 fold respectively, in treated compared to untreated cells (**Figure 3C**). In addition, increased VEGF production and KDR protein expression was demonstrated by ELISA measurement and biochemical analysis of HUVEC lysates, respectively. As shown in **Figure 3D**, quantitative measurements of the secreted protein revealed a 1.7-fold increase of VEGF in conditioned media from L-NAME treated cells in comparison to untreated cells. Similarly, KDR protein level in cells chronically treated with L-NAME is 1.8-fold increased as compared to untreated cells (**Figure 3E**), in good agreement with the RT-qPCR data.

A major VEGF signaling pathway involved in endothelial cell migration includes the activation of phosphatidylinositol-3-kinase (PI-3-K) and the subsequent phosphorylation of the protein kinase AKT, which in turn activates eNOS by phosphorylating it on Ser1177. To determine if L-NAME treatment modified the ability of VEGF to activate this pathway, the phosphorylation state of eNOS and AKT after a 5-min VEGF stimulation in control and chronically L-NAME treated cells was measured. As shown in **Figure 3F**, in control cells VEGF (25 ng/ml) increased eNOS and AKT phosphorylation by about 3 times, as expected (lane 2). In L-NAME treated cells, the basal levels of eNOS and AKT phosphorylation were already increased (see lane 3 *vs* lane 1), and VEGF was not able to induce any further phosphorylation (lane 4). A densitometric analysis performed on 4 independent experiments revealed that in L-NAME treated cells the basal level of phosphorylated eNOS was 3.43±0.94 times greater than in control cells. The increase was less pronounced when the basal level of phosphorylated AKT was compared in treated and control cells (1.57±0.24 times).

The results presented in Figs. 3 C-F are consistent with an activated VEGF/KDR system in L-NAME-treated HUVECs, and could explain the enhancement of both basal and VEGF-stimulated chemotactic response in these cells.

### Inhibition of basal NO production induces nuclear HIF-1α accumulation in HUVECs

**Figure 4:**
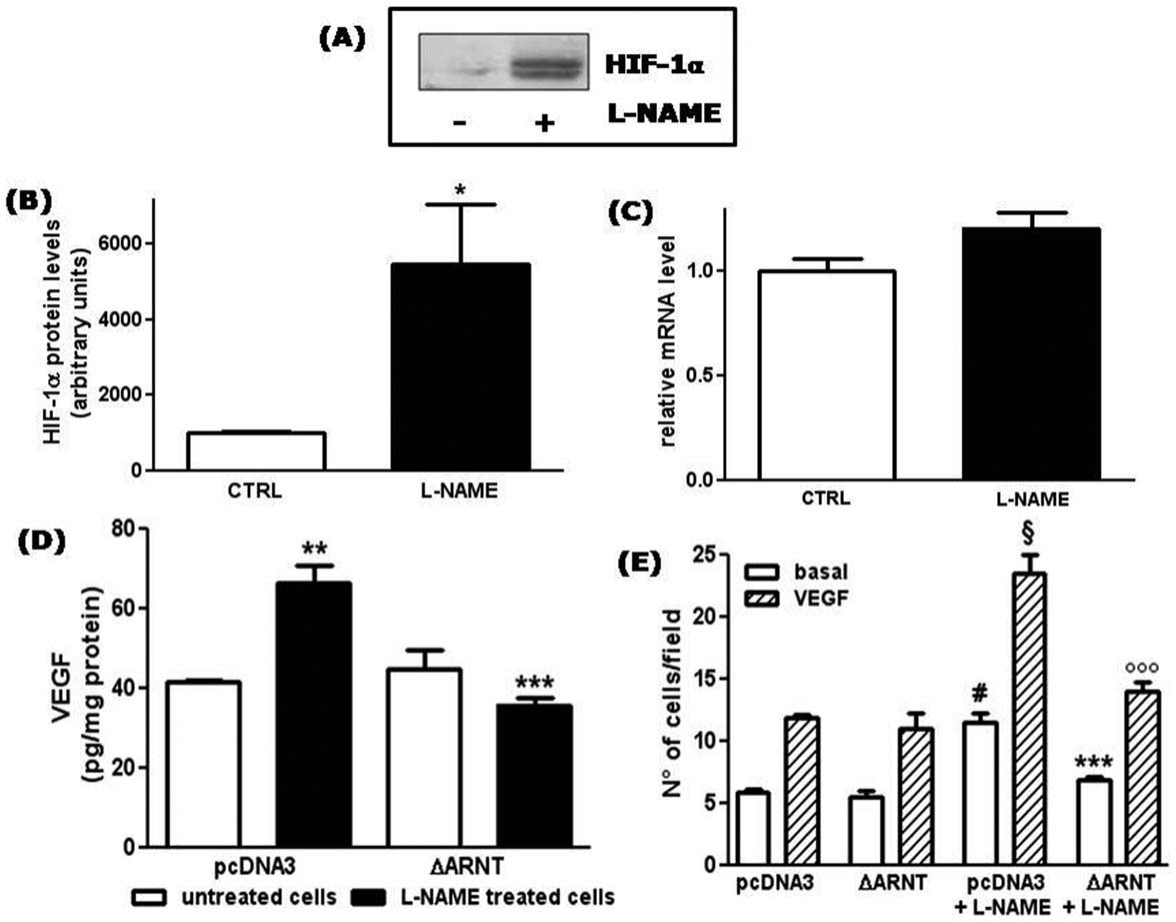
L-NAME treatment induces HIF-1α nuclear accumulation. (A) HIF-1α protein levels were detected by western blotting of nuclear extracts from control HUVECs (lane 1) or from HUVECs treated with L-NAME for 48 h (lane 2). Shown is a representative blot of 4 comparable experiments. HIF-1α migrates as a doublet with apparent molecular weight of 118 and 120 kDa. (B) Densitometric analysis of nuclear HIF-1α protein levels. *p<0.05; *t* test; n = 4. (C) HIF-1α RNA levels were measured by RT-qPCR and normalized to the level of the housekeeping gene 18S. No significant differences between control and L-NAME treated cells (*t* test; n = 3). (D) VEGF protein levels were detected by ELISA measurement in conditioned media collected from HUVECs transfected with the empty vector (pcDNA3) or with the expression vector ΔARNT, and treated with L-NAME for the 48 h following transfection. Results are expressed as pg of VEGF normalized to the cell protein content (pg/mg protein). **p<0.01 *vs* untreated cells transfected with pcDNA3; ***p<0.001 *vs* L-NAME treated cells transfected with pcDNA3 (One-way ANOVA with Bonferroni's test; n = 3). (E) HUVECs were transfected with pcDNA3 or ΔARNT, and treated with L-NAME for the 48 h following transfection when indicated. Chemotaxis experiments were then performed using 25 ng/ml VEGF as attractant. Results are expressed as the number of migrating cells. #p<0.001 *vs* basal migration in untreated pcDNA3 cells; §p<0.001 *vs* VEGF-induced migration in untreated pcDNA3 cells; ***p<0.001 *vs* basal migration in pcDNA3 cells treated with L-NAME; °°°p<0.001 *vs* VEGF-induced migration in pcDNA3 cells treated with L-NAME; no significant differences between untreated pcDNA3 and ΔARNT transfected cells and between untreated and L-NAME treated ΔARNT tranfected cells (One-way ANOVA with Bonferroni's test, n = 10).

Increased VEGF production and cell motility are typical events occurring in hypoxic cancer cells, due to the accumulation of hypoxia-inducible factor-1α (HIF-1α), which plays a major role in the transcriptional activation of genes encoding angiogenic factors [18], [19]. Similarly, induction of VEGF expression during hypoxia has been described also in endothelial cells [20]. We therefore analysed the effect of long term L-NAME treatment on HIF-1α levels in HUVECs. Most interestingly, we observed that, after 48 h of treatment, L-NAME induced nuclear accumulation of HIF-1α in HUVECs (5.5±1.6 fold over basal) (**Figures 4A and B**). RT-qPCR analysis revealed no significant change in HIF-1α mRNA levels after L-NAME treatment (1.21±0.1 fold in comparison to untreated cells) (**Figure 4C**), suggesting that HIF-1α accumulation in L-NAME-treated cells was mainly due to its stabilization, as occurs under hypoxic conditions. Taken together, these data suggest that prolonged L-NAME treatment induces in endothelial cells a pseudohypoxic state, with the consequent stabilization of HIF-1α under normoxic conditions.

In addition to HIF-1α, other factors could contribute to the up-regulation of VEGF expression [21]. To directly correlate the increase in VEGF expression induced in HUVECs by L-NAME treatment to the transcriptional activity of HIF-1, we performed experiments in which L-NAME treated cells and control cells were transfected with a plasmid expressing a dominant negative form of the HIF-1β subunit (ΔARNT), which maintains the capacity of forming an heterodimer but cannot bind DNA [22], [23]. As shown in **Figure 4D**, in HUVECs, the increase in VEGF protein induced by L-NAME treatment was totally blunted, confirming the central role of HIF-1α as regulator of VEGF expression in NO-deprived endothelial cells. Moreover, as shown in **Figure 4E**, transfection with ΔARNT inhibited also the increased migratory capacity induced in HUVECs by L-NAME treatment.

### The NO donor DETA-NO reverts the effects of L-NAME treatment on HIF-1α stabilization, and on VEGF and eNOS expression

**Figure 5:**
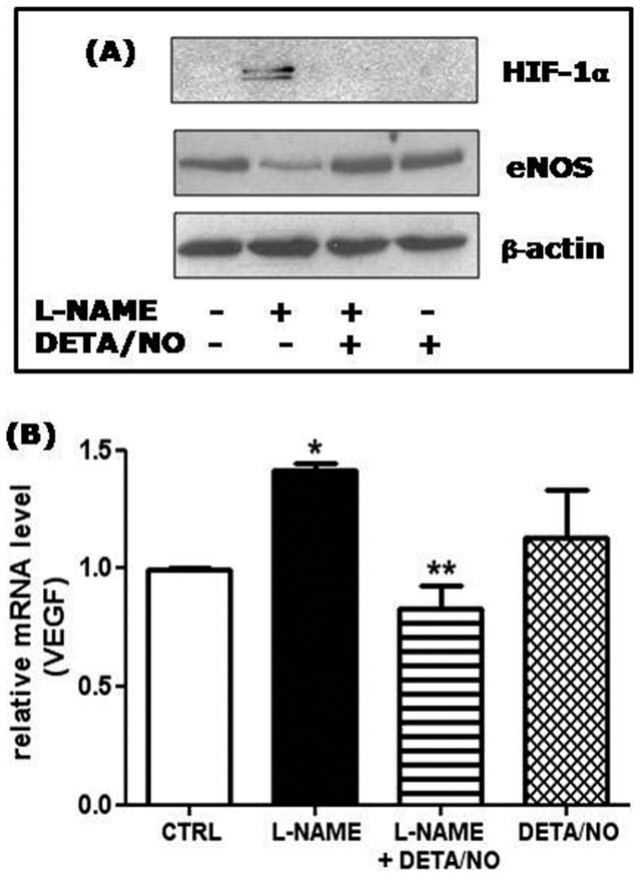
The NO donor DETA-NO reverts the effects of L-NAME treatment on HIF-1α stabilization, and on VEGF and eNOS expression. (A) HIF-1α protein levels were detected by western blotting of nuclear extracts from HUVECs treated with L-NAME and/or DETA/NO as described in Fig. 2A. An aliquot of total cell lysates was immunoblotted with anti eNOS antibodies, and with anti β-actin antibodies as loading control. A representative blot of 3 comparable experiments is shown. (B) VEGF RNA levels were measured by RT-qPCR and normalized to the level of the housekeeping gene 18S. *p<0.05 *vs* control cells (CTRL); **p<0.01 *vs* L-NAME treated cells; no significant differences between control and DETA/NO treated cells (One-way ANOVA with Bonferroni's test; n = 3).

To investigate whether the observed effects of L-NAME treatment on HUVEC gene expression were due to the chronic depletion of NO, we reconstituted NO levels by utilizing the long lasting NO donor DETA-NO. The addition of 500 nM DETA-NO completely reverted the stabilization of nuclear HIF-1α, the reduced expression of total eNOS protein (**Figure 5A**) and the increase in VEGF mRNA levels (**Figure 5B**), which are all characteristic features detected in the long-term L-NAME treated cells.

### Silencing of eNOS mimics the effects of long-term L-NAME treatment of HUVECs

**Figure 6:**
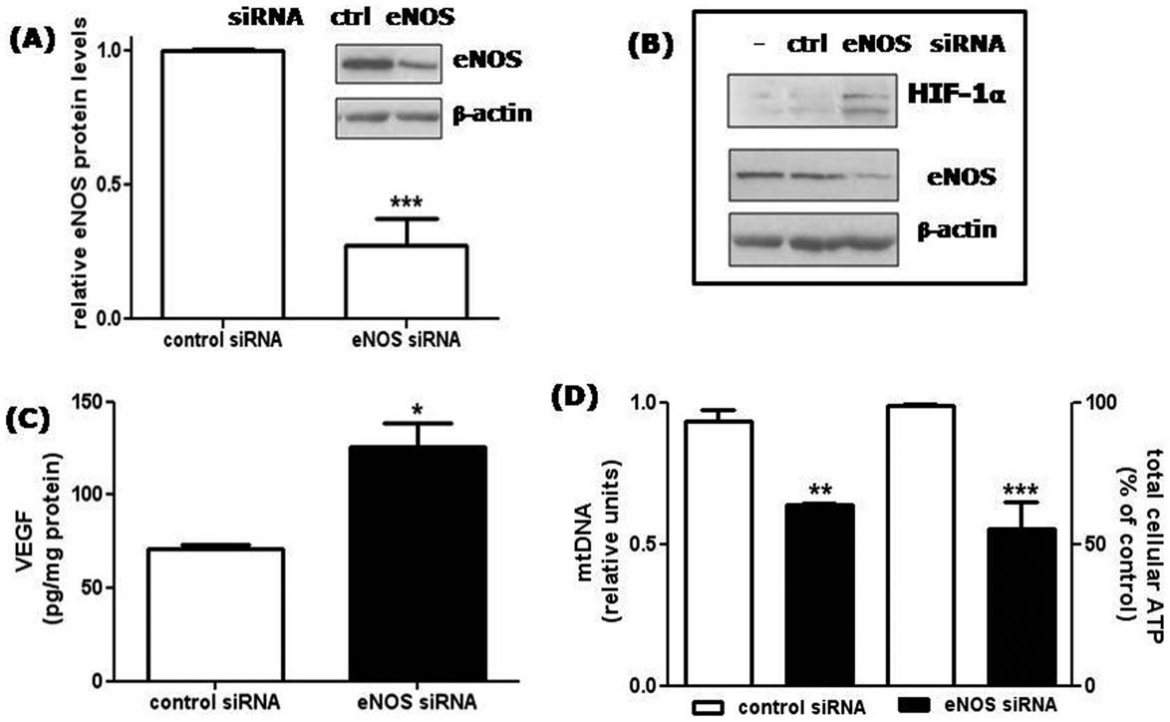
Effect of eNOS silencing on HIF-1α accumulation, VEGF secretion, mtDNA and ATP levels. (A) Characterization of HUVECs transfected with eNOS siRNA: densitometric analysis of eNOS protein expression where eNOS protein levels were normalized to β-actin protein. ***p<0.001; *t* test; n = 4. Inset: representative blots of eNOS protein in cells transfected with control (ctrl) or eNOS siRNA. (B) HUVECs were transfected with control (lane 2) or eNOS siRNA (lane 3), and HIF-1α protein was detected by western blotting on the corresponding nuclear extracts. In lane 1, nuclear extracts from untransfected cells. An aliquot of total cell lysates was immunoblotted with anti eNOS antibodies to check silencing, and with anti β-actin antibodies as loading control. A representative blot of 2 comparable experiments is shown. (C) VEGF protein levels were detected by ELISA measurement in conditioned media collected from HUVECs 48 h after transfection with control or eNOS siRNA. Results are expressed as pg of VEGF normalized to the cell protein content (pg/mg protein). *p<0.05; *t* test; n = 3. (D) MtDNA (left axis) and total cellular ATP content (right axis) were measured in HUVECs transfected for 48 h with control or eNOS siRNA. In silenced cells, mtDNA and ATP were reduced by 36±0.4 and 45±9.7% respectively. **p<0.01 and ***p<0.001; *t* test; n = 3.

To further investigate if the observed effects of L-NAME treatment were due to the specific inhibitory effect on eNOS activity, expression of the enzyme was silenced in HUVECs by using RNA interference methodology. HUVEC transfection with eNOS siRNA caused a mean reduction in eNOS protein levels of 70±0.1% (**Figure 6A**)**.** In agreement with the results obtained after long-term exposure to L-NAME, HIF-1α accumulated in the nucleus of eNOS knock-down cells, whereas cells transfected with control siRNA were unaffected (**Figure 6B**). Moreover, in eNOS silenced HUVECs, again similarly to long term L-NAME treated cells, VEGF production was increased (**Figure 6C**), while the amount of mtDNA as well as ATP levels were decreased (**Figure 6D**).

## Discussion

Our results demonstrate that long-term pharmacologic and genetic down-regulation of eNOS induce relevant effects in HUVECs. The long-term treatment with L-NAME induced a significant decrease in mtDNA amount, accompanied by decreased mitochondrial activity and ATP production. This observation is in agreement with previous results, which showed the important role of the gas in promoting mitochondrial biogenesis in different cell types and tissues [16], [17]. Similar results (*i.e*. decreased mitochondrial mass) were obtained in the microvasculature of mice treated with N*^G^*-monomethyl-L-arginine (L-NMMA) for a long period of time (1–2 months) [24]; however, given the length of the treatment, these results are not directly comparable to ours. To our knowledge, the effect of NO deprivation for shorter times (days) on mitochondrial biogenesis in ECs had not previously investigated. Our work demonstrated that HUVECs respond to NO deprivation already after 48 h of treatment, making them a useful model for the investigation of the effects of the gas on mitochondrial biogenesis and function.

An important consequence of the decrease in NO production observed by us is the accumulation of HIF-1α in the nucleus, with a resulting adaptation of gene expression to a pseudohypoxic state, *i.e.* a state of hypoxia in the presence of normal levels of oxygen. The increased expression of VEGF found in our experiments can be considered a consequence of this pseudohypoxic state since the dominant negative form of the HIF1-β subunit ΔARNT totally blunted the VEGF protein increase induced by L-NAME treatment. Accordingly, ΔARNT transfection inhibited also the increased motility in L-NAME treated HUVECs, thus indicating that the migratory behavior we observed in the treated cells can be considered the consequence of the transcriptional activity of HIF-1α. Of note, the increased HUVEC motility is observed despite a reduction in eNOS levels, and is not induced by chronic treatment with the guanylate cyclase inhibitor ODQ, indicating that cGMP and protein kinase G (PKG) are not involved in the observed effects.

Our results open the interesting question of the mechanism by which NO deficiency induces HIF1αstabilization in HUVECs. HIF-1α degradation depends on prolyl hydroxylases-catalysed proline hydroxylation, which induces binding of the factor to an ubiquitin ligase (the Von-Hippel Lindau protein) and targets it for proteasomal degradation. The activity of prolyl hydroxylases (PHDs) is dependent on the availability of oxygen and 2-oxoglutarate (a Krebs cycle intermediate) as substrates and on Fe^2+^ and ascorbate as cofactors. In addition to these mechanisms controlling HIF levels, HIF-1α transcriptional activity can also be negatively controlled via hydroxylation of an asparaginyl residue operated by an O_2_ dependent enzyme called Factor Inhibiting HIF (FIH-1) [25]. Under hypoxic conditions, PHD and FIH-1 activity are inhibited and HIF-1α accumulates in the nucleus to function as a transcription factor and to evoke adaptive responses to changes in tissue oxygenation. Under our experimental conditions, chronic eNOS inhibition induces HIF-1α nuclear accumulation, apparently as a result of a decreased degradation, supporting the hypothesis of an impaired PHD activity. The question is then shifted to the mechanism through which NO may affect PHD activity in HUVECs.

It is well known that NO competes with O_2_ for the binding to the heme moiety of cytochrome C oxidase [26]–[28]. Therefore, a possibility that we considered was that NO deficiency, by activating mitochondrial respiration, could cause an intracellular O_2_ redistribution from the cytoplasm to the mitochondria [29], thus inducing oxygen depletion and PHD inactivation in the cytosol. However, it is unlikely that cytoplasmic hypoxia could have occurred in our experimental conditions where cells are exposed to an atmospheric O_2_ concentration (21% O_2_) [30]. In addition, in NO deficient cells we observed a lower oxygen consumption, which can be explained by the reduced mitochondrial mass, thus making the hypothesis of an O_2_ redistribution from the cytoplasm to the mitochondria difficult to support. We conclude therefore that NO deficiency affects PHD activity under normoxic conditions.

The relationship between NO and HIF-1α is complex and matter of intense debate [31]. Recent studies have suggested a dual role for NO in regulating HIF-1α function. By using NO donors or the controlled expression of an inducible NOS, it has been found that high concentrations of NO induced HIF-1α nuclear accumulation, even under normoxic conditions, whereas under hypoxic conditions, when HIF-1α levels are already high, low physiological concentrations of NO appear to have the opposite effect [32]–[37]. The effects of the gas under the latter conditions are similar to those observed here. Further underscoring the similarity, the involvement of soluble guanylate cyclase and cGMP in the low NO-induced reduction of HIF-1α was, like in our migration experiments, excluded [32]–[34], [38]. However, differently from the previously reported results, in our experiments, the ECs were not subjected to hypoxic conditions. It is possible that in ECs, the O_2_ threshold for HIF-1α stabilization is higher than in other cells, and that under normoxic conditions, basal NO production counteracts the stabilization that would otherwise occur.

It should be also considered that PHD activity is affected not only by O_2_, but also by the availability of iron, ascorbate and the Krebs cycle intermediate 2-oxoglutarate [39]. The deficiency of NO may influence the intracellular availability of one or more of these factors, thus decreasing the enzymatic activity and causing HIF-1α accumulation. We cannot however exclude that other stabilizing mechanisms, not directly linked to PHD, are involved [40]. Furthermore, the possibility that reactive oxygen species may play a role is now under investigation in our laboratory. Certainly, more work is needed to clarify these important points.

In our experiments very low concentrations of DETA-NO abrogated the HIF-1α accumulation, the VEGF increased expression and the increased motility found in L-NAME treated cells. On the basis of this result one can hypothesize that low levels of the gas constantly produced by ECs under basal conditions contribute to keep HIF-1α levels under control. In other words, under physiological conditions, the ECs, by producing constitutive NO, could control all of the events set in motion by HIF-1α, including cell motility and VEGF production. In case of NO deficiency, HIF-1α would be released from this brake, would escape degradation and consequently accumulate, as shown in our experiments.

In conclusion, our results show that lack of NO in human endothelial cells induces pseudohypoxia and mitochondrial dysfunction with consequent decreased energy production. These events may very well occur in all of those pathological conditions where eNOS expression and/or activity are impaired, as in hypertension, type 2 diabetes, hypercholesterolemia, thus contributing to the endothelial dysfunction typical of such disorders. Our experimental model where eNOS activity was impaired by pharmacological and genetic inhibition may represent a good *in vitro* system to study the effect of NO deficiency on vascular endothelial cells, and to find new therapeutic target (HIF-1α protein for example) possibly useful for treating endothelial dysfunction.

## Materials and Methods

### Cell cultures

Human umbilical vein endothelial cells (HUVECs) were isolated from freshly derived umbilical cords by digestion with collagenase as described by Jaffe et al. [41]. Umbilical cords were donated anonymously after informed consent according to national ethical legislation. Cells were routinely grown in 199 medium, supplemented with 20% heat-inactivated fetal bovine serum (FBS), 100 µg/ml endothelial cell growth supplement (ECGS) and 50 µg/ml heparin, and used at passages 2–7. Where indicated, HUVECs were treated with 5 mM *N* ^G^-Nitro-L-arginine methyl ester (L-NAME) in 199 medium containing 10% FBS for 48 h preceding the experiments. The concentration of L-NAME was chosen according to Papapetropoulos et al. [10].

### Apoptosis assays

Quantification of apoptosis/necrosis was performed by Annexin V-FITC conjugate and propidium iodide (PI) staining (Abcam, Cambridge, UK) followed by fluorescence activated cell sorting (FACS) performed with a FACScalibur flow cytometer equipped with a 488 nm argon laser (Becton Dickinson, San José, CA, USA). The collected data were evaluated by Cell Quest software. The degree of apoptosis was calculated as apoptotic index considering cells both in early and late apoptosis. In addition, active caspase-3, Bcl-2 and Bax proteins were detected by western blot on total cell lysates.

### Evaluation of mitochondrial DNA (mtDNA)

Total DNA was extracted with QIAamp DNA extraction kit (Qiagen, Hilden, Germany), and mtDNA levels were amplified with primers specific for the mitochondrial cytochrome *b* (CytB) gene and normalized to genomic DNA by amplification of the rRNA 18S nuclear gene. Primers used were: for CytB, F, 5′–cttcgctttccacttcatcttacc-3′ and R, 5′-ttgggttgtttgatcctgtttcg-3′, and for 18S, F, 5′-ctgccctatcaactttcgatggtag-3′ and R, 5′-ccgtttctcaggctccctctc-3′. Quantitative real time PCR (RT-qPCR) reactions were run with the iQ SybrGreenI SuperMix (Bio-Rad, Segrate, Italy) on an iCycler iQ Real-Time PCR detection system (Bio-Rad) using 50 ng of total DNA. Calculations were performed with the 2^−ΔΔCt^ methods using 18S rRNA as an internal control.

### Oxygen consumption

Cellular oxygen consumption was measured as previously described [42]. Briefly, HUVECs were re-suspended in respiration buffer (0.3 M mannitol, 10 mM KCl, 5 mM MgCl_2_, 10 mM K_2_PO_4_, pH 7.4) [43] at a density of 3.0×10^6^/ml, and analyzed at 37°C in a gas-tight vessel equipped with a Clark-type oxygen electrode (Rank Brothers Ltd., Cambridge, UK) connected to a chart recorder. Protein content in cell samples was determined by the bicinchoninic acid (BCA) protein assay (Thermo Scientific, Rockford, IL, USA).

### Cell metabolism assays

Cell metabolism was assessed by means of a Cell Titer 96® Aqueous ONE Solution Reagent colorimetric assay (MTS, Promega, Madison, WI, USA), and the total cellular ATP content using a CellTiter-Glo® Luminescent Assay (Promega). Both the assays were performed according to the manufacturer's instructions on HUVECs plated at a density of 2×10^4^ cells/well in 96-well microplates. Optical density at 490 nm (for MTS) and luminescence (for ATP) were measured using a multiplate spectrophotometer (Victor™, PerkinElmer, Waltham, MA, USA). On parallel wells, cell proliferation was evaluated by crystal violet staining. Briefly, cells were fixed with 100% methanol, and then stained with a 0.1% crystal violet solution. After extensive washes with deionized water, the dye was solubilized in 10% acetic acid solution, and the absorbance was measured at 595 nm.

### Cell migration assays

HUVEC migration was evaluated by means of chemotaxis experiments in a 48-well modified Boyden chamber, as previously described [13], [14]. Briefly, the filters coated with 10 µg/ml of type IV collagen were placed over a bottom chamber containing 25 ng/ml VEGF as attractant factor. The cells, suspended in 199 media containing 2% FBS, were added to the upper chamber at a density of 5.0×10^4^ cells/well. After 6 h of incubation at 37°C, the cells that had migrated to the lower side of the filter were stained with Diff-Quick stain (VWR Scientific Products, Bridgeport, NJ, USA), and 5 unit fields per filter were counted by a scorer blind to the experimental conditions using a Zeiss microscope. The assays were run in triplicate.

### Determination of cGMP accumulation

HUVECs were cultured in 60-mm Petri dishes and treated for 48 h with L-NAME or ODQ. During the last 30 min of treatment, 1 mM isobutylmethylxanthine (IBMX) was added to inhibit phosphodiesterases. cGMP was extracted in 500 µl of 0.1 N HCl, and its quantification was performed by an enzyme immunoassay (EIA) kit (Enzo Life Sciences, Vinci-Biochem, Vinci, Firenze, Italy) following manufacturer's instructions for the acetylated assay procedure.

### Immunoblot and immunoprecipitation analyses

For immunoblot analysis, HUVECs, plated in 35-mm diameter Petri dishes, were washed with phosphate-buffered saline (PBS), and then directly lysed in SDS-PAGE sample buffer (62 mM Tris-HCl pH 6.8, 2% sodium dodecyl sulfate (SDS), 10% glycerol, 5% 2-mercaptoethanol, and 0.04% bromophenol blue). After SDS-PAGE electrophoresis, proteins were transferred onto nitrocellulose membranes that were blocked with 5% (w/v) non fat dried milk in Tris-buffered saline containing 0.05% Tween-20 (TBS-T), and probed overnight with the indicated primary antibodies. After incubation with the appropriate peroxidase-conjugated secondary antibody (DAKO, Denmark), the immunoreactive bands were visualized by chemioluminescence (LiteAblot Plus, EuroClone, Italy). For KDR immunoprecipitation, HUVECs were washed with PBS and lysed for 10 min on ice with RIPA buffer (20 mM Tris-HCl pH 7.5, 150 mM NaCl, 1 mM EDTA, 1 mM EGTA, 1% NP-40, 1% sodium deoxycholate, 0.1% SDS, 1 mM β-glycerophosphate, 1 mM sodium fluoride, 1 mM sodium orthovanadate) supplemented with protease inhibitors. Aliquots of cleared cell lysates containing the same amount of protein (250 µg/sample) were incubated with anti KDR antibodies, followed by incubation with Protein A Sepharose. The immune complexes were washed with lysis buffer, eluted by boiling in sample buffer, and analyzed by SDS-PAGE. Densitometric analyses of the immunoblots were performed using the National Institute of Health (NIH) Image J program.

### ELISA determination of VEGF levels

Cell supernatants were collected from HUVECs plated in 35-mm Petri dishes, and VEGF measurements were performed using commercially available ELISA kits (R&D Systems, Minneapolis, MN, USA) following manufacturer's instructions. VEGF levels were expressed relative to total cell protein (pg/mg of total protein) evaluated by BCA protein assay.

### Preparation of nuclear extracts

HUVECs, plated in 100-mm diameter Petri dishes, were washed with PBS and collected by scraping. The cells were then lysed for 10 min at 4°C in buffer A (10 mM HEPES pH 8.0, 1.5 mM MgCl_2_, 10 mM KCl, 0.5 mM dithiothreitol (DTT), 0.05% Nonidet P-40, 1 mM sodium orthovanadate) supplemented with protease inhibitors. After a 10 min centrifugation at 2,500 *g* at 4°C, the crude nuclei were washed with buffer A prior to lysis in buffer C (20 mM HEPES pH 8.0, 1.5 mM MgCl_2_, 420 mM NaCl, 1.0 mM DTT, 0.2 mM EDTA, 1 mM sodium orthovanadate, supplemented with protease inhibitors) for 30 min at 4°C. The nuclear extracts were clarified by centrifugation, and loaded on a 10% SDS-PAGE. The immunoreactive bands were visualized by chemioluminescence after overnight incubation with the anti HIF-1α antibody at a 1∶500 dilution in 5% BSA in TBS-T.

### Total RNA extraction for reverse transcription and quantitative real time PCR

Total RNA was extracted using the RNeasy® Mini Kit and accompanying QIAshredder™ (Qiagen, Hilden, Germany) according to the manufacturer's instructions. To avoid DNA contamination of samples, a 15 min on column incubation was carried out with DNase I (Qiagen). Reverse transcription was performed using the SuperScript^™^ III First-Strand Synthesis System for RT-PCR (Invitrogen), again following the manufacturer's instructions. For quantitative analysis of gene expression we used the ABI Prism® 7000 Sequence Detection System, SDS software version 1.2.3 (Applied Biosystems, CA, USA). Target sequences were amplified from 50 ng of cDNA. TaqMan® Primer and Probe assays used were: human VEGF-A (Hs99999070_m1), KDR (Hs00176676_m1), eNOS (NOS3, Hs00167166_m1), HIF-1A (Hs00153153_m1) and the endogenous control 18S (Hs99999901_s1). For calculation of results, the 2^−ΔΔCt^ method was used allowing normalization to 18S and to the calibrator which is set to a value of 1.

### Transient transfection

HUVECs, plated in 35-mm Petri dishes, were transfected with the expression vector pcDNA3ARNTdelta_b (ΔARNT) (kindly provided by Dr. Tacchini, Department of Human Morphology and Biomedical Sciences ‘Città Studi’, University of Milan, Milano, Italy), coding for a dominant negative mutant form of the HIF-1β ARNT subunit, and the void vector pcDNA3 using *Trans*ITTM LT1 (Mirus, Bologna, Italy). Six hours after transfection, the culture medium was replaced by fresh medium, and the cells were exposed to L-NAME for the following 48 h.

### Small interfering RNA (siRNA) transfection

Validated Stealth^TM^ RNAi duplexes against human eNOS (GC content 48%) were provided from Invitrogen. As control RNA, we utilized a Stealth^TM^ RNAi negative control duplex (Medium GC Duplex, Invitrogen) with a 48% GC content, suitable for use as a control with Stealth^TM^ RNAi duplexes containing 45-55% of GC. All sets of RNAi molecules were transfected individually into HUVECs at a 30 nM concentration using Lipofectamine 2000 according to the manufacturer's instructions (Invitrogen). The ability of the RNAi molecules to knockdown eNOS expression was analyzed 48 h after transfection by western blot analysis.

### Statistical procedures

All data were expressed as mean ± s.e.m. Statistical analysis was carried out using one-way analysis of variance (ANOVA) followed by Bonferroni's multiple comparison test or Student's *t*-tests, where applicable.

### Reagents and antibodies

All tissue culture reagents were from Sigma Chemicals (St. Louis, MO, USA). The following reagents were purchased as indicated: human VEGF_165_ from Calbiochem (Darmstadt, Germany); collagen type IV from BD Bioscience (Bedford, MA, USA); L-NAME, ODQ (1H-[1], [2], [4]oxadiazolo[4,3-a]quinoxalin-1-one), DETA-NO (2,2′ (hydroxynitrosohydrazono)bis-ethanimine) from Sigma Chemicals (St. Louis, MO, USA).

Antibodies used were: rabbit polyclonals anti caspase-3 (Cell Signalling, Beverly, MA, USA), anti Bax and anti KRD (Santa Cruz Biotechnology, Santa Cruz, CA, USA), and mouse monoclonals anti Bcl-2 (Santa Cruz Biotechnology), anti eNOS, anti HIF-1α (BD Transduction Laboratories, Franklin Lakes, NJ, USA) and anti β-actin (Sigma Chemicals, St. Louis, MO, USA).
